# Clinical features and potential markers of disease in idiopathic non-histaminergic angioedema, a real-life study

**DOI:** 10.1007/s12026-024-09501-9

**Published:** 2024-06-03

**Authors:** Ilaria Mormile, Maria Celeste Gigliotti, Anne Lise Ferrara, Roberta Gatti, Giuseppe Spadaro, Amato de Paulis, Stefania Loffredo, Maria Bova, Angelica Petraroli

**Affiliations:** 1 Department of Translational Medical Sciences, University of Naples Federico II, Via Sergio Pansini 5, Naples, 80131 Italy; 2 Center for Basic and Clinical Immunology Research (CISI), WAO Center of Excellence, University of Naples Federico II, Naples, Italy; 3 Post-Graduate Program in Clinical Immunology and Allergy, University of Naples Federico II, Naples, Italy; 4 Institute of Experimental Endocrinology and Oncology “G. Salvatore” (IEOS), National Research Council (CNR), Naples, Italy; 5 Department of Internal Medicine, A.O.R.N. Antonio Cardarelli, Naples, Italy

**Keywords:** Acquired angioedema, Bradykinin, C1 inhibitor, Hereditary angioedema, Icatibant, Idiopathic non-histaminergic acquired angioedema, omalizumab

## Abstract

Idiopathic non-histaminergic acquired angioedema (InH-AAE) is a rare disease, with unknown etiology and pathogenesis, characterized by recurrent clinical manifestations and resistance to antihistamines and corticosteroids. We aim to evaluate clinical features and potential markers of disease in an Italian cohort of patients with InH-AAE. We enrolled 26 patients diagnosed with InH-AAE. Information about clinical features, treatments, routine laboratory investigations, immunological and genetic tests were collected. We assessed plasma levels of complement components, angiogenic and lymphangiogenic mediators, proinflammatory cytokines and chemokines, and activity of phospholipases A2. Finally, patients underwent nailfold videocapillaroscopy (NVC); both quantitative and qualitative capillaroscopic parameters were analyzed. Plasma levels of VEGFs were similar in healthy controls and in InH-AAE patients. ANGPT1 was decreased in InH-AAE patients compared to controls while ANGPT2 was similar to controls. Interestingly, the ANGPT2/ANGPT1 ratio (an index of vascular permeability) was increased in InH-AAE patients compared to controls. sPLA2 activity, elevated in patients with C1-INH-HAE, showed differences also when measured in InH-AAE patients. TNF-α concentration was higher in InH-AAE patients than in healthy controls, conversely, the levels of CXCL8, and IL-6 were similar in both groups. At the NVC, the capillary loops mainly appeared short and tortuous in InH-AAE patients. InH-AAE represents a diagnostic challenge. Due to the potential life-threatening character of this condition, a prompt identification of the potentially bradykinin-mediated forms is crucial. A better comprehension of the mechanism involved in InH-AAE would also lead to the development of new therapeutic approaches to improve life quality of patients affected by this disabling disease.

## Introduction

Recurrent angioedema can present with or without wheals [1]. Angioedema without wheals may be driven by bradykinin and/or mast cell mediators. The bradykinin-mediated forms include the hereditary forms (HAE), the acquired C1-esterase inhibitor deficiency angioedema (C1-INH-AAE), and the angioedema related to angiotensin-converting enzyme inhibitor therapy (ACEi-AAE) [2]. In bradykinin-mediated angioedema, several organs and systems may be affected, including the skin, gastrointestinal tract [3], upper airways [4], and urinary and genital tracts [5]. Other rare manifestations include neurologic [6] and psychologic/psychiatric symptoms [7, 8]. Several factors may trigger the attacks, including emotional stress, physical trauma, and invasive medical procedures [9–11].

In about one-third of patients presenting with recurrent angioedema, no underlying cause can be identified; these patients are consequently diagnosed with idiopathic angioedema (IAE) [12]. The 2021 revision and update of the international WAO/EAACI guideline for the management of hereditary angioedema classifies mast cell mediator-induced AE in IgE mediated and non-IgE mediated, and identifies the idiopathic forms as the conditions in which the mediator is unknown [2]. This classification represents an update of the largely accepted classification by Cicardi et al. [13] which further stratified IAE patients according to the response to antihistamines, dividing idiopathic angioedema in idiopathic histaminergic acquired angioedema (IH-AAE) in those presenting a good response to these drugs, and idiopathic non-histaminergic acquired angioedema (InH-AAE), in patients with persistent recurrences of symptoms despite prophylaxis with continuous doses of antihistamines [2, 13–15]. In this article, InH-AAE designates forms of recurrent angioedema that are not hereditary with persistent recurrences upon antihistamine treatment, excluding other known causes of acquired angioedema. This type of bradykinin-mediated angioedema is more severe than mast cell-mediated forms, with an estimated 45-fold higher risk of death [16]. Although IH-AAE is the most common form of angioedema without urticaria, seen in 96% of consultations [17], some patients with IH-AAE do not respond to a fourfold dose of antihistamine. Of interest, the efficacy of the anti-IgE omalizumab in InH-AAE suggests that mast cells have a primary role in a subset of patients with InH-AAE [10]. Mast cells degranulation can also trigger the activation of factor XII (FXII) and lead to the generation of bradykinin through the release of heparin, tryptase, and elastase or other mediators [18]. In 1999, Cicardi et al. [19] used for the first time the term InH-AAE to describe a group of patients who had similar clinical features and responded to prophylactic treatment with tranexamic acid (TXA) as patients affected by C1-IHN-HAE suggesting similar pathogenesis in the two angioedema forms. In addition, the bradykinin B2 receptor-targeted drug icatibant seems to be effective in reverting symptoms in InH-AAE patients [20–23].

InH-AAE patients probably represent the most heterogeneous population in the angioedema landscape and several pathogenetic aspects involved in the development of the disease remain largely unknown. In addition, literature is currently limited and consensus guidelines for the management of this condition are still missing. This article aims to describe clinical features and treatment patterns in an Italian cohort of patients with InH-AAE. We also aim to evaluate vessel characteristics through nailfold videocapillaroscopy (NVC) and assess their possible correlation with plasma levels of vascular permeability factors and cytokines.

## Methods

### Patients

26 patients diagnosed with InH-AAE were enrolled. Inclusion criteria were age ≥ 18 years, history of angioedema for at least one year, unresponsiveness to fourfold dose of antihistamine for 1–3 months, normal C3, C4, C1-INH antigenic levels and functional activity, negative genetic tests for HAE (i.e., *SERPING1, F12, PLG, ANGPT1, KNG1*, and *MYOF*), available data on sex, date of birth, age of onset and signature of the written informed consent. Exclusion criteria were the presence of urticaria, use of ACEi drugs previously or at the onset of symptoms, and concomitant autoimmune diseases.

All data were collected from February 2020 and November 2023.

At the enrollment, information about comorbidities, medications, and routinary, and disease-specific laboratory investigations (i.e., complete blood count, liver function tests, kidney function tests, LDH, glucose, lipid panel, serum tryptase, and total IgE) were collected. During the follow-up, semestral visits were scheduled, and participants were asked about attack frequency, duration, localization, trigger factors (i.e., physical trauma, emotional distress, menstruation cycle, food ingestion, drugs, infections, exposure to cold/heat, pressure, or vibration), and rescue medications used. 26 and 10 healthy Caucasian age-, gender-, and Body Mass Index (BMI)-matched subjects without angioedema were chosen as controls for plasma levels of vascular permeability factors and cytokines and NVC respectively. The controls had been referred for a routine medical check-up. All participants were excluded if they had concomitant autoimmune diseases (e.g., systemic lupus erythematosus, systemic sclerosis, arthritis, and Raynaud phenomenon) and comorbidities potentially impacting the NVC outcome (e.g., arterial hypertension, diabetes mellitus, and hypo/hyperthyroidism).

**Fig. 1:**
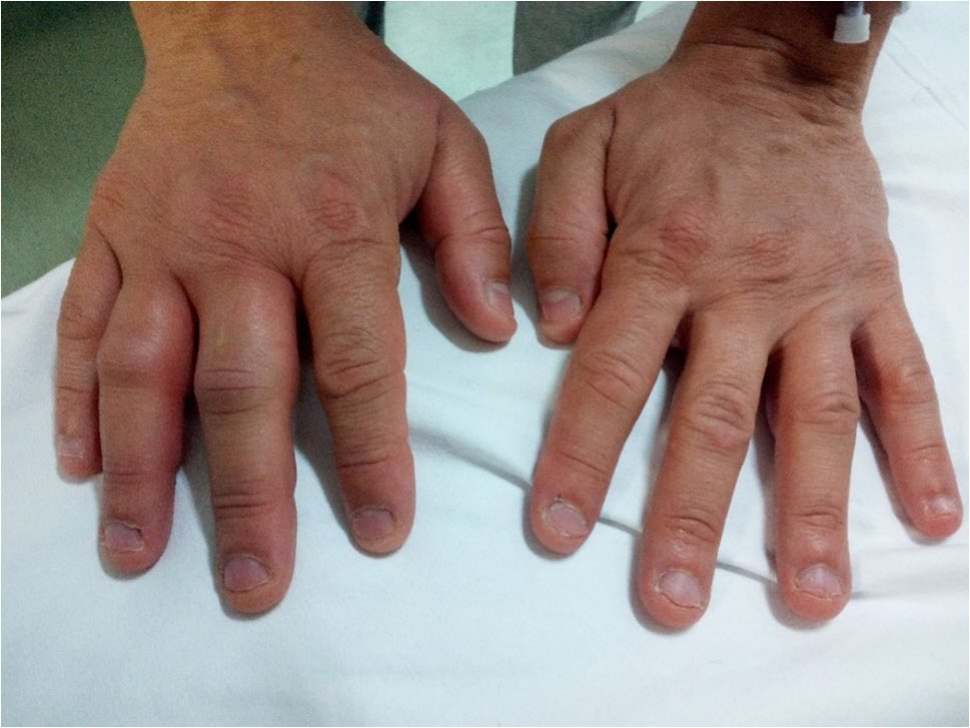
Photograph of the right hand angioedema taken by a patient

This study protocol was approved by the Ethics Committee of the University Hospital Federico II of Naples, Italy (Protocol n. 1553/18). All the subjects enrolled gave informed consent to participate in the study. An informed written consent was obtained for the use of human images (Fig. 1).

### Plasma collection

Blood was collected during routine diagnostic procedures, and the plasma sample was labeled with a code, which was entered into a datasheet. The controls had been referred for a routine medical check-up and gave their informed consent to participate. The technicians who performed the assays were blind to the patients’history. The samples were collected by means of venipuncture and minimal stasis using 3.2% sodium citrate. After centrifugation (2000 g for 20 min at 22 °C), the plasma was divided into aliquots and stored at − 80 °C until usage. Blood samples from all patients were obtained at least 8 days apart from an angioedema attack (remission sample).

### Determination of mediators by ELISA

Plasma levels of angiogenic/lymphangiogenic and immunomodulating mediators were measured using commercially available enzyme-linked immunosorbent assay (ELISA) kits for vascular endothelial growth factor-A (VEGF-A), VEGF-A165b, VEGF-C, VEGF-D, angiopoietin 1 (ANGPT1), ANGPT2, Tumor Necrosis Factor-α (TNF-α), interleukin (IL)-6, CXCL8 (R&D System, Minneapolis, Minnesota, USA) according to the manufacturer’s instructions. The sensitivity of ELISA is 31.1–2000 pg/mL for VEGF-A, 62.5–4000 pg/mL for VEGF-A165b, 62.5–4000 pg/mL for VEGF-C, 31.1–2000 pg/mL for VEGF-D, 156.25-10 000 pg/mL for ANGPT1, 31.1–4000 pg/mL for ANGPT2, 15.6–1000 pg/ml for TNF-α, 9.38–600 for IL-6 and 31.25–2000 for CXCL8.

### PLA_2_ activity assay

Activity of phospholipases A2 (PLA_2_) in plasma of patients and healthy controls was measured by Life Technologies EnzChek®phospholipase A_2_ assay. Briefly, a PLA_2_ substrate cocktail consisting of 7-hydroxycoumarinyl-arachidonate (0.3 mM), 7-hydroxycoumarinyl-linolenate (0.3 mM), hydroxycoumarinyl 6-heptenoate (0.3 mM), dioleoylphosphatidylcholine (DOPC) (10 mM), and dioleoylphosphatidylglycerol (DOPG) (10 mM) was prepared in ethanol. Liposomes were formed by gradually adding 77 µl substrate/lipid cocktail to 10 ml of PLA_2_ buffer (50 mM Tris–HCl, 100 mM NaCl, 1 mM CaCl2) while stirring rapidly over 1 min using a magnetic stirrer Fluorescence (excitation at 360 nm and emission at 460 nm) was measured and specific activity [relative fluorescent units (RFU)/ml] for each sample was calculated. Plasma (50 µl) of patients and healthy controls was added to 96-well plates, and PLA_2_ activity was evaluated by adding 50 µl of substrate cocktail.

### Complement system

C1-INH and C4 antigen levels were measured by means of radial immunodiffusion (RID) (NOR-Partigen, Siemens Healthcare Diagnostics, Munich, Germany). C1-INH function was assessed as the capacity of plasma to inhibit the esterase activity of exogenous C1s as measured on a specific chromogenic substrate by means of a commercially available kit (Technoclone GmbH, Vienna, Austria). Reference ranges were: 0.70 to 1.30 Unit C1-INH/ml (1 Unit C1-INH corresponds to the average C1-INH activity present in 1 ml of fresh citrated normal plasma). The functional activity of C1-INH was also expressed as a percentage of activity of C1-INH present in samples. Normal values of activity of C1-INH are greater than 0.7 Unit C1 INH/ml (> 70%). All patients enrolled in this study showed a C1-INH functional activity > 50%, as previously reported [24].

### Nailfold Videocapillaroscopy

NVC is a non-invasive diagnostic procedure for the in vivo study of the structural characteristics of nailfold small vessels [25]. This investigation is performed by placing a microscope combined with a digital video camera on the nailfold [25].

All InH-AAE patients underwent the procedure during remission (at least eight days after an attack). The exam was performed using the CapillaryScope VideoCap 3.0-D1 (DS Medica, Milan, Italy), by the same single experienced operator to reduce operator bias. The study staff performing/scoring the procedure were blinded to patient group. Before performing NVC, patients remained at rest for at least 15 min at a set temperature of 20–22 °C to reduce the influence of climate on the exam outcome (e.g., false positive for avascular zone). Both hands and 2nd-5th fingers were examined, excluding thumbs as suggested by most scholars due to the poor-quality images usually seen at this level [26, 27]. Cedar oil was applied at the nailfold to enhance the transparency of the epidermis. Fingers with localized trauma were avoided to minimize false positive patterns (e.g., hemorrhages, abnormal shapes).

A global evaluation was performed with ×200 magnification high-resolution objective [27]. At least four images for each finger were captured (i.e., two lateral and two medial fields).

Both quantitative and qualitative capillaroscopic parameters were analyzed. The quantitative parameters were:

- capillary density (n/mm): number of capillaries in a 1-mm length of the distal row of the nailfold (reference range: 7–12 capillaries/mm) [26, 27];
- intercapillary distance (µM): distance between two neighboring capillary loops, measured at the widest intercapillary space in the central capillary region [26, 27];
- apical diameter (µM): distance from one external margin of the capillary loop to another on the apex (normal, 8–20µM; dilated capillaries, > 20µM and < 50 µM; or giant capillaries, > 50 µM) [26, 27];
- internal diameter (µM): the distance between the efferent and the afferent loop measured at the same level [26, 27];
- external diameter (µM): the width of a capillary at its widest Sects. [26, 27];
- loop length (µM): the distance between the apex of a capillary loop and the point where the capillary is no longer visible [26, 27].

The qualitative parameters analyzed were:

- capillary distribution: organization of capillaries, scored as ordered (0), comma-like (1), irregular (2), and severely deranged (3);
- capillary morphology: the shape of capillaries, scored as hairpin-like (0), mainly tortuous (1; afferent and efferent limbs bend but do not cross, once crossing shape, twice crossing shape), mainly ramified (2; branching, bushy or coiled capillaries), severe alteration (3; meandering capillaries, bizarre capillaries) [26, 27];
- microhemorrhages: the absence (0) or presence (1) of extra-capillary brown aggregates.

of clotted blood [28];

- interstitial edema: scored as absence (0) or presence (1) of fluid accumulation in the interstitial space [29].

### Statistic analysis

Data were analyzed with the GraphPad Prism 5 software package. Data were tested for normality using the D’Agostino-Pearson normality test. If normality was not rejected at 0.05 significance level, we used parametric tests. Otherwise, for not-normally distributed data we used nonparametric tests. Statistical analysis was performed by unpaired two-tailed t-test or two-tailed Mann-Whitney test as indicated in figure legends. Correlations between two variables were assessed by Pearson ‘s correlation analysis and reported as coefficient of correlation (*r*). A *p* value ≤ 0.05 was considered statistically significant. Plasma levels of VEGFs, ANGPTs, cytokines and chemokines are shown as the median (horizontal black line), the 25th and 75th percentiles (boxes) and the 5th and 95th percentiles (whiskers) of 26 controls and 26 InH-AAE patients. Capillaroscopic parameters are shown as the median (horizontal black line), the 25th and 75th percentiles (boxes), and the 5th and 95th percentiles (whiskers) of 10 controls and 12 InH-AAE patients. Statistically significant differences were accepted when the *p* value was ≤ 0.05.

## Results

### Demographics and clinical features

26 adult patients diagnosed with InH-AAE were enrolled. Clinical features in our cohort of patients are summarized in Table 1.

**Table 1:** Clinical features in our cohort of patients with idiopathic non-histaminergic angioedema (n°26)

Patients’ features	
Female gender (n, %)	11 (42.30%)
Caucasian ethnicity (n, %)	26 (100%)
Age, years (mean ± SD, range)	50 ± 15 (17–70)
Age at onset (mean ± SD, years)	36 ± 18.75 (5–75)
Attack frequency (≥ 12 attacks/year; n, %)	15 (57.69%)
Average attack duration:	
<6 h (n, %)	0 (0%)
6–12 h (n, %)	5 (19.23%)
13–24 h (n, %)	5 (19.23%)
25–48 h (n, %)	8 (30.76%)
>48 h	8 (30.76%)
Attack localization:	
Skin (n, %)	25 (96.15%)
Gastrointestinal tract (n, %)	5 (19.23%)
Tongue (n, %)	11 (42.30%)
Larynx (n, %)	14 (53.84%)
Genitalia (n, %)	8 (30.76%)

11 out of 26 (42.30%) patients were affected by the following allergic diseases: allergic rhinitis (*N* = 5; 9.23%), adverse drug reaction (i.e., non-steroidal anti-inflammatory drugs and antibiotics) (*N* = 3; 11.53%), atopic dermatitis (*N* = 2; 7.69%), and asthma (*N* = 1; 3.84%). All these comorbidities were in good clinical control. Routine laboratory investigations were within the reference range for all patients.

Attacks occurrence and their characteristics were also investigated (Table 1). With reference to attack frequency, 15 out of 26 patients (57.69%) reported ≥ 12 attacks per year; 7 out of 26 patients (26.92%), and 4 patients (15.38%) presented with less of a single attack per year. Mean attack frequency was 11.08 ± 7.84.

16 (61.53%) patients identified a trigger factor for the attacks. Most frequently reported trigger factors were emotional distress (*n* = 11; 42.30%), physical trauma (*n* = 8; 30.76%), and drugs (*n* = 7, 26.92%). Non-steroidal anti-inflammatory drugs were the most common drugs associated with angioedema attacks. Other trigger factors for the attacks were menstruation cycle (*n* = 2, 7.69%), food ingestion (*n* = 2, 7.69%) and physical stimuli such as exposure to cold/heat, pressure, or vibration (*n* = 2, %).

14 (53.84%) out of 26 patients referred to the emergency department for at least an angioedema attack.

All patients were asked about previous surgeries. 15 (57.69%) out of 26 patients underwent surgery and only one patient had an angioedema attack following the surgical procedure.

All patients have negative genetic tests for known mutations up to date for HAE (i.e., *SERPING1, F12, PLG, ANGPT1, KNG1*, and *MYOF*). One patient presented with the heterozygous mutation IVS4 ds + 31 G > A in the *SERPING1* gene intronic variant. This mutation has been previously described in the literature in a patient with C1-INH-HAE [30] and has been included in the Human Genome Mutation Database (https://www.hgmd.cf.ac.uk/ac/index.php). Despite that, the patient did not show decreased C1-INH levels (0.378 g/l; reference range 0.21–0.39) nor C4 level (0.23 g/l; reference range 0.10–0.40). C1-INH functional activity was 106% (reference range > 50%). The patient’s clinical features were negative familiar history, late onset of disease (first episode of swelling occurring when she was 57 years old), and absence of abdominal attacks. In this case, the correlation between the patient’s genotype and phenotype appears not univocal since the mutation did not determine a reduction of C1-INH level or impairment of its functional activity. Therefore, we did not classify the patient as HAE and included her in this study.

Data about on-demand treatment and long-term prophylaxis (LTP) were collected (Table 2). Both pdC1-INH and icatibant were effective in controlling the attacks in all patients.

**Table 2:** Treatment strategies in our cohort of patients with idiopathic angioedema (n°26)

Therapy for acute attacks	
pdC1-INH(n, %)	1 (3.8%) ^a^
Icatibant (n, %)	8 (30.76%) ^a^
LTP	
Patients treated with LTP (n, %)	12 (46.15%)
TXA (n, %)	3 (11.53%)
Attenuated androgens (n, %)	0 (0%)
pdC1-INH (n, %)	1 (3.8%)
Lanadelumab (n, %)	2 (7.69%)
Omalizumab (n, %)	6 (23.07%)
pdC1-INH, plasma-derived C1-inhibitor; LTP, Long-term prophylaxis; TXA, tranexamic acid. ^a^one patient was provided with both pdC1-INHand icatibant as on-demand therapy for acute attack.	

Omalizumab was the most used treatment for LTP (*n* = 6; 23.07%). Patients were treated with omalizumab for a mean duration of 22.83 months (1–60 months). In all patients, omalizumab was effective in controlling attack recurrences, and no adverse effect was observed. 3 patients (11.53%) were treated with TXA for an average period of 36 months with good clinical outcome. 2 patients (7.69%) were treated with off-label lanadelumab 300 mg administrated subcutaneously every two weeks for 24 months with an optimal control of symptoms.

### Plasma concentrations of VEGFs and ANGPTs in idiopathic angioedema patients

**Fig. 2:**
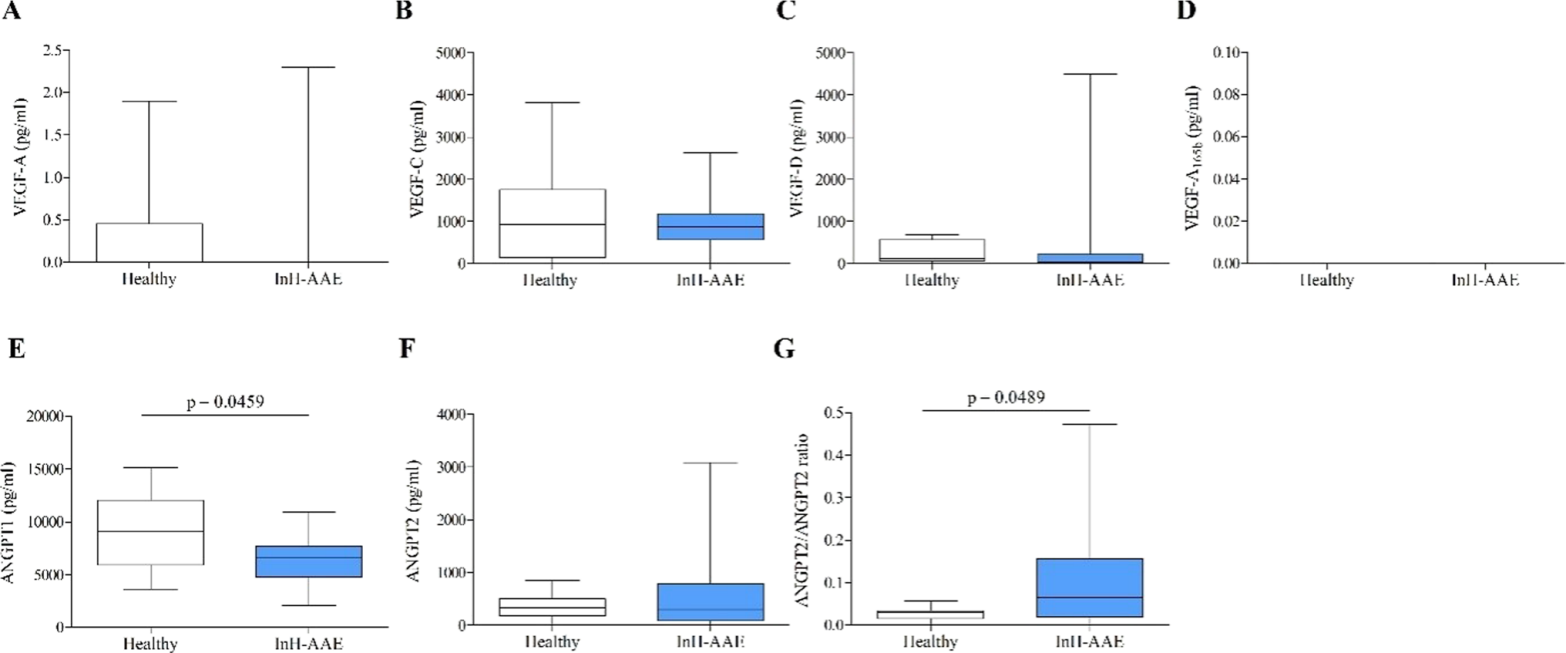
Plasma VEGF-A (A), VEGF-C (B), VEGF-D (C) and VEGF-A_165b_ (D), ANGPT1 (E), ANGPT2 (F), ANGPT2/ANGPT1 ratio (G) in controls (Healthy) and patients with idiopathic non-histaminergic acquired angioedema (InH-AAE) in remission. Median (horizontal black line), 25th and 75th percentiles (boxes) and the 5th and 95th percentiles (whiskers) of 26 controls and 26 patients

We evaluated the concentrations of different angiogenic and lymphangiogenic factors in InH-AAE patients in remission. Figure 2 shows that VEGF-A (panel A), VEGF-C (panel B) and VEGF-D (panel C) plasma levels of InH-AAE patients were not different to that of healthy controls. The VEGF-A gene encodes for several splicing variants, such as VEGF-A_165a_ and VEGF-A_165b_ [31, 32]. Similar results were obtained when we observed VEGF-A_165b_ that prevents the VEGF-A_165a_ effects on increased vascular permeability, vasodilatation, and angiogenesis [32]. The plasma level of the VEGF-A_165b_ was not increased in InH-AAE patients compared to controls (panel D).

ANGPT1was decreased in InH-AAE patients compared to controls [Figs. 1E and 6.6 (4.7–7.7) vs. 9.1 (5.8–12.1) ng/ml median values (interquartile ranges)] while ANGPT2 was similar to controls (Fig. 2F). The Ang2/Ang1 ratio [33] was increased in InH-AAE patients compared to controls [Fig. 2G; ANGPT2/ANGPT2 ratio: 0.07(0.02–0.16) vs. 0.03 (0.01–0.03) ng/ml median values (interquartile ranges)].

There was no difference in VEGFs and /or ANGPTs concentration between male and female values in both controls and patients (data not shown). Moreover, the age of patients and the concentration of the different plasma mediators examined did not correlate (data not shown).

### Plasma concentrations of proinflammatory mediators in idiopathic angioedema patients

**Fig. 3:**
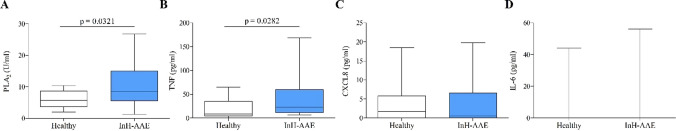
Plasma PLA_2_ (A), TNF-α (B), CXCL8 (C), and IL-6 (D) in controls (Healthy) and in patients with idiopathic non-histaminergic acquired angioedema (InH-AAE) in remission. Median (horizontal black line), the 25th and 75th percentiles (boxes) and the 5th and 95th percentiles (whiskers) of 26 controls and 26 patients

sPLA_2_ activity, elevated in patients with C1-INH-HAE [34], showed differences also when measured in InH-AAE patients. In particular, Fig. 3A shows that InH-AAE patients had higher activity of sPLA_2_ compared to healthy controls [8.4 (5.5–14.9) vs5.7(3.6–8.6) U/ml median values (interquartile ranges)]. Interestingly, the concentrations of these mediators did not differ between symptomatic and asymptomatic FXII-HAE patients (data not shown). Next, we measured the plasma level of proinflammatory cytokines and chemokines in InH-AAE patients. Figure 3B shows that TNF-α concentration was higher in InH-AAE patients than in healthy controls [22.6 (11.4–60.4) vs. 8.5 (4.6–35.2) U/ml median values (interquartile ranges)]. Conversely, the levels of CXCL8 and IL-6 in InH-AAE patients were similar that of controls (Fig. 3C, D).

### Capillaroscopic parameters in idiopathic angioedema patients

**Fig. 4:**
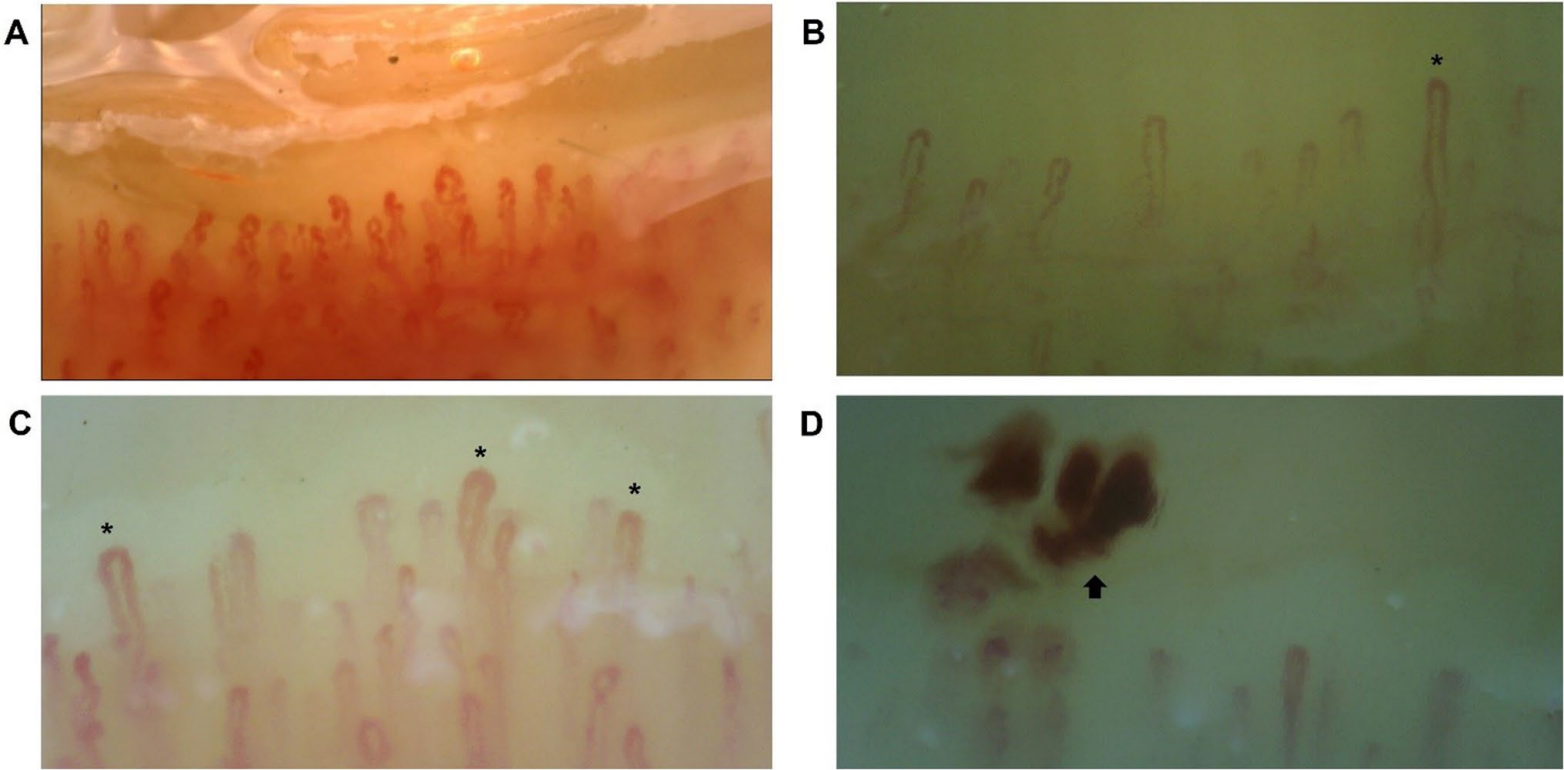
Images from nailfold videocapillaroscopy on recruited idiopathic non-histaminergic acquired angioedema (InH-AAE) patients. Tortuosity and decreased loop length (A). Dilated capillary (*) with apical diameter > 20 mm and < 50 mm and normal “hairpin” shapes (B, C). Presence of single and multiple (arrow) pericapillary microhemorrhages and marked interstitial edema (D). ×200 magnification

We have previously demonstrated that C1-INH-HAE patients had altered capillaroscopic parameters [25]. In a final series of experiments, we checked whether InH-AAE patients had the same capillaroscopic alterations found in C1-INH-HAE patients. Twelve InH-AAE patients (Figs. 4) and 10 healthy controls underwent NVC.

**Fig. 5:**
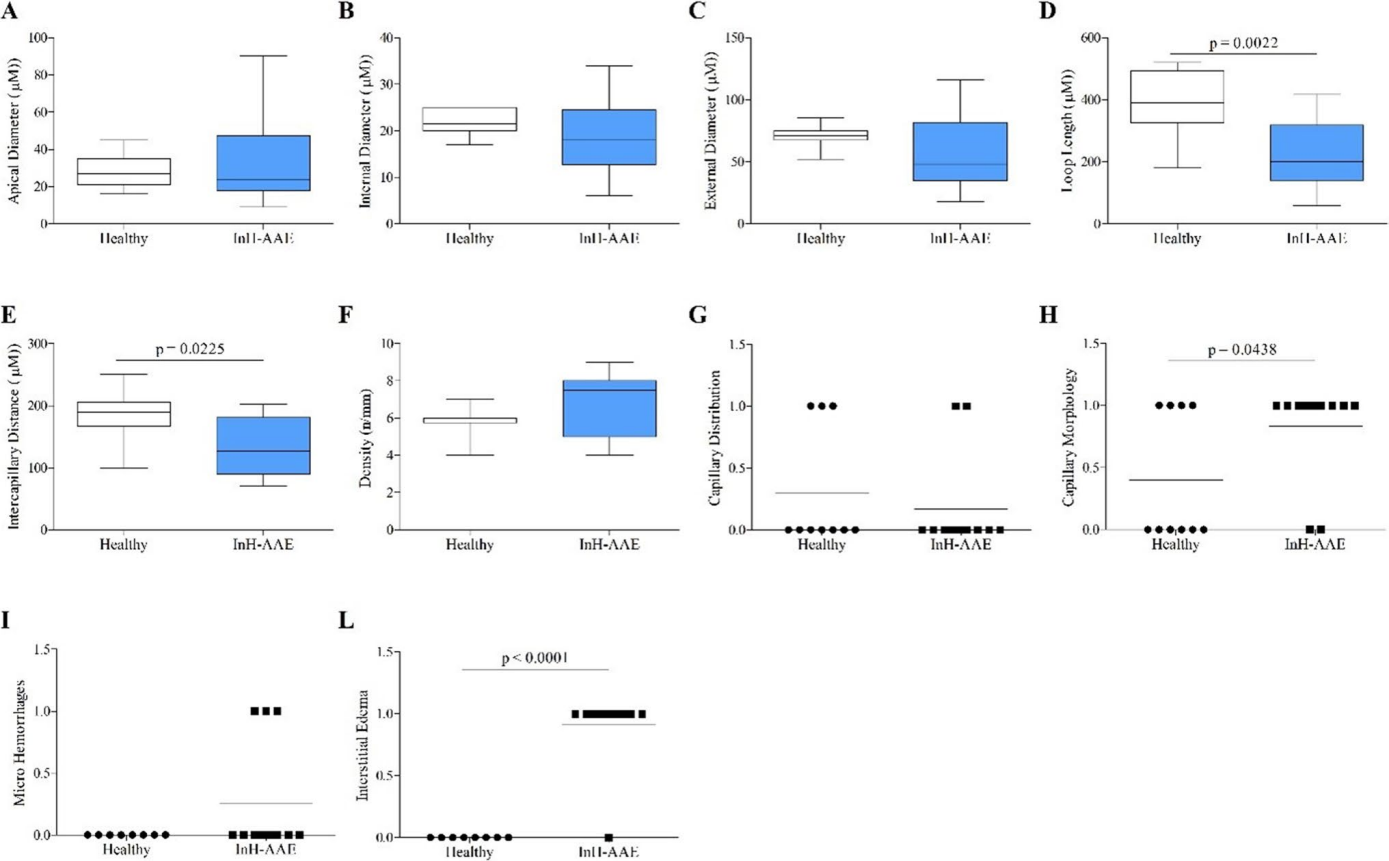
Capillaroscopic parameters (A-L) in 13 controls (Healthy) and 13 patients with idiopathic non-histaminergic acquired angioedema (InH-AAE) in remission. Horizontal bars depict the median value (A-L), boxes the 25th and 75th percentiles, and whiskers the 5th and 95th percentiles (A-F)

Apical (Fig. 5A), internal (Fig. 5B) and external diameters (Fig. 5C) were similar between InH-AAE patients and healthy controls. Conversely InH-AAE patients showed significantly decreased of loop length (Fig. 5D) and intercapillary distance (Fig. 5E). In addition, the capillary density (Fig. 5F) and distribution (Fig. 5G) were similar in InH-AAE patients and controls. No avascular areas (absence of adjacent capillaries) were found in both patients and controls.

Capillary morphology was mainly tortuous (Fig. 5H) in InH-AAE patients versus controls. The number of micro hemorrhages in InH-AAE patients was not altered but they presented more interstitial edema (Fig. 5L) compared to controls. Although tortuous shapes were common among patients, no capillary ramifications or meandering capillaries denoted as “abnormal” capillary morphology according to the EULAR Study Group consensus framework [27] were observed.

## Discussion

Bradykinin-mediated angioedema is often a diagnostic challenge due to its overlap with the more common allergic conditions [35] and other diseases [36, 37], leading the patients to see multiple healthcare specialists enhancing the diagnostic delay. Especially for the idiopathic form, there is a lack of consensus regarding the diagnostic and therapeutic management [10]. Some reports have shown the efficacy of omalizumab in InH-AAE [38, 10, 39–41], as well as in chronic urticaria with or without angioedema resistant to antihistamines [42–45]. Due to the lack of response to antihistamines in InH-AAE, bradykinin has been postulated as the major mediator in the pathogenesis of the disease. However, the successful response to omalizumab suggests the concomitance of a mast cell-mediated process [10]. The underlying pathogenetic mechanisms in InH-AAE largely remain to be elucidated. For this reason, the choice between long-term treatment with omalizumab rather than other prophylactic drugs active on the bradykinin pathway represents an unmet need in clinical practice. Indeed, some authors have reported a successful symptom control for both on-demand therapy and LTP with drugs regulating bradykinin action in some patients with InH-AAE patients (i.e., pdC1-INH, icatibant, ecallantide, TXA, and lanadelumab) [19–21, 46–48]. Of interest, the promising kallikrein inhibitor lanadelumab is under investigation for LTP also in patients with non-histaminergic angioedema with normal C1-INH (NCT04206605, NCT04444895). These results would possibly provide further information about the use of this drug in patients with less understood forms of bradykinin-mediated angioedema. In our cohort, both icatibant and pd-C1INH were effective as on-demand therapy. In addition, two patients started an off-label LTP with lanadelumab with a marked reduction of the attack frequency.

**Fig. 6:**
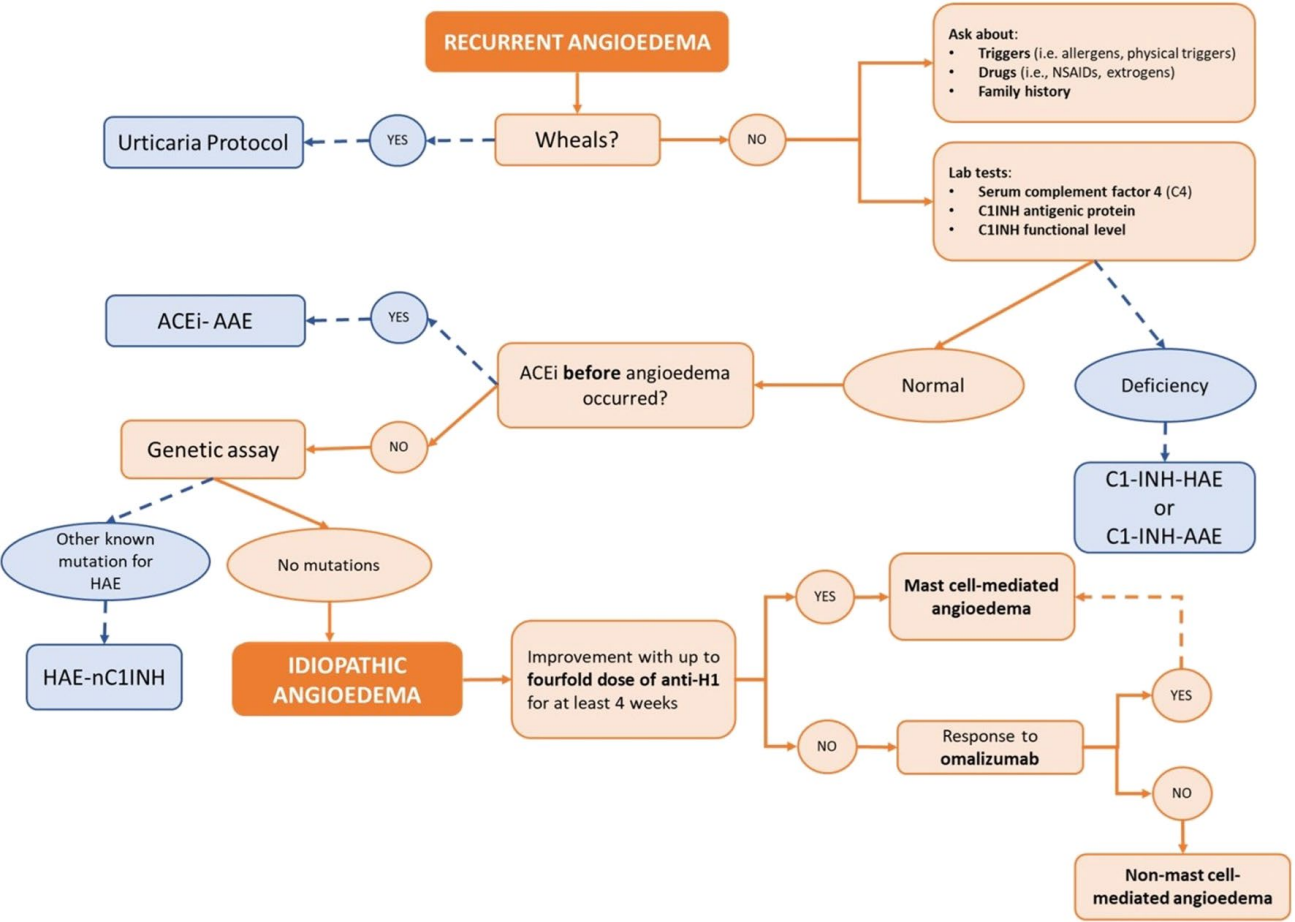
Diagnostic flowchart for recurrent angioedema. ACEi-AAE, angioedema related to angiotensin-converting enzyme inhibitor therapy; C1-INH, C1-esterase inhibitor; C1-INH-AAE, acquired C1-esterase inhibitor deficiency angioedema; C1-INH-HAE, hereditary angioedema due to C1-esterase inhibitor deficiency; HAE, hereditary angioedema; HAE-nC1-INH, hereditary angioedema with normal C1 inhibitor; NSAID, non-steroidal anti-inflammatory drugs

The response to certain drugs could also be used in the diagnostic workup as an indirect marker of the angioedema subtype. In Fig. 6 we propose a feasible protocol based on our experience and the existent literature to guide physicians in the approach to the patient with recurrent angioedema with a focus on the idiopathic forms. InH-AAE should be suspected after having ruled out all the other tentative diagnoses in patients experiencing the recurrence of angioedema without wheals despite the continuous administration of a 4-fold antihistamine dose for at least four weeks [49, 50]. Other characteristic clinical features of bradykinin-mediated angioedema are the duration of the attacks, which usually last over 24 h, and the peculiar involvement of other organs and systems such as the gastrointestinal tract [49]. However, even in the case of antihistamine unresponsiveness, a non-specific mast cell activation cannot be excluded. For this reason, a trial with omalizumab could be appropriated [38, 10, 39–41].

Literature about NVC findings in recurrent angioedema is scarce. A recent work by Mostmans et al. [51] conducted in patients with chronic spontaneous urticaria (67.4% of patients with recurrent angioedema in addition to wheals) showed significantly lower capillary density, more capillary malformations, and more irregular capillary dilations on NVC in patients as compared to controls. Another case report by Tsuzuki et al. [52] described self-limiting NVC abnormalities including micro-bleeding and abnormal morphologies in a patient with angioedema with eosinophilia, even though, in this last case, the NVC alterations could be due to the well-known effects of eosinophils on microcirculation [53] rather to recurrencies of angioedema itself. Our group previously evaluated vascular features through NVC in patients with C1-INH-HAE, discovering significant structural capillary alterations [25]. We found that C1-INH-HAE patients showed greater intercapillary distance, increased apical, internal, and external diameter, decreased density, irregular capillary distribution, and more tortuous morphology [25]. In the same study, we also analyzed NVC features in a cohort of patients with ACEi-AAE, observing no qualitative or quantitative alterations compared to hypertensive controls [25]. Based on these findings, we hypothesized that capillary alteration may be typical of the hereditary forms, aligning with the endothelial dysfunction described in these patients [54, 55]. In the present article, we decided to further shed light on this topic by analyzing features of nailfold capillaries in patients affected by InH-AAE. Interestingly, no variation in the capillary diameter compared to the control group was found. In addition, the intercapillary distance, which usually correlates with the capillary density, was decreased in InH-AAE (Fig. 5E), suggesting that capillary density is not impaired in InH-AAE patients. Moreover, in InH-AAE we observed a significantly decreased loop length (Fig. 5D), so the capillary loops mainly appeared short and tortuous (Fig. 5H). Differences in capillaroscopic patterns among the different angioedema subtypes should be evaluated on larger cohorts to assess if some findings could be considered specific for a particular angioedema form. Indeed, we cannot exclude that the variation in the frequencies of some alterations could be due to the small sample examined in our study. Indeed, our study is subject to some limitations, including the single-center design and the small sample size. However, this data could be a starting point to possibly correlate the capillaroscopic pattern observed in these patients with different angioedema subtypes, possibly adding this exam in the diagnostic work-up.

In order to shed lights on the possible involvement of microcirculation in the pathogenesis of bradykinin-mediated angioedema we previously compared 128 patients with C1-INH-HAE and 68 healthy controls demonstrating in the patient group higher plasma levels of angiogenic factors (VEGF-A, VEGF-C, Ang1, and Ang2) [33]. These mediator levels were also significatively higher in patients with ≥ 12 attacks/year [33]. Hence, we hypothesized that VEGFs and ANGPTs can induce a state of ‘vascular preconditioning’ that might predispose to angioedema attacks in patients with C1-INH-HAE [25, 33, 56]. This hypothesis was further corroborated by the major structural capillary alterations we found in C1-INH-HAE [25]. In another study, we also evaluated VEGFs, ANGPTs and secreted PLA_2_ during the acute attack compared to remission in 15 patients with C1-INH-HAE [56]. VEGFs and ANGPT2 levels were not altered, while ANGPT1, a vascular stabilizer, were increased during attacks compared to symptoms-free periods [56]. In addition, the ANGPT2/ANGPT1 ratio (an index of vascular permeability) was decreased during angioedema attacks [56]. In this study, we found that plasma levels of VEGFs were not different between InH-AAE patients and healthy controls (Fig. 2). Interestingly, ANGPT1, which inhibits vascular permeability, was decreased in InH-AAE patients compared to controls while ANGPT2, that is a vascular destabilizer, was similar to controls. These finding did not align with the results we observed in C1-INH-HAE patients, possibly suggesting that a major disfunction in microcirculation could be more typical of the hereditary angioedema. On the other hand, the Ang2/Ang1 ratio was increased in InH-AAE patients compared to controls [33]. We cannot exclude that this apparently contrasting observations could be due to the greater heterogeneity of InH-AAE patients compared to C1-INH-HAE population.

In addition, InH-AAE patients had higher activity of PLA_2_ (Fig. 3A) similarly to patients with C1-INH-HAE [34]. PLA_2_ family can directly modulate vascular permeability and endothelial cell migration in vitro [56–58]. In turn, the production of these enzymes by endothelial cells is inducted by bradykinin [59]. Interestingly, it was previously reported that the activity of PLA_2_ is decreased during angioedema attacks [34]. The mechanism underlying this observation is still unclear, even though it could be possibly due to their exhaustion or sequestration during the angioedema attacks [60]. In this view, PLA_2_ levels could be considered a further indicator of the involvement of the bradykinin-mediated pathway in our cohort, even if further studies are needed to validate the use of this mediator on a broader scale.

TNF-α concentration was higher in InH-AAE patients than healthy controls (Fig. 3B). Data about the role of this cytokine in InH-AAE are currently missing. However, it has been reported that TNF-α augments activation of the prekallikrein-high molecular weight kininogen complex to generate kallikrein and bradykinin [61]. Moreover, this cytokine can induce the release of urokinase, which can convert plasminogen to plasmin and represents a possible source for bradykinin generation [61]. In addition, a recent article by Gramstad et al. [62] investigated the thromboinflammatory load in C1-INH-HAE, reporting significantly higher TNF levels in patients as compared to controls. According to the authors HAE this observation may reflect a subclinical attack state beside the acute swelling episodes.

In conclusion, InH-AAE still represents a diagnostic challenge, and validated guidelines for choosing tailored treatments are an unmet need. However, due to the potential life-threatening character of recurrent angioedema, a prompt identification of the potentially bradykinin-mediated form to adapt the treatment choice is crucial. A better comprehension of the mechanism involved in the idiopathic form of angioedema would also lead to the development of new therapeutic approaches to improve the quality-of-life patients affected by this disabling condition.

## Data Availability

The data presented in this study are available on request from the corresponding author.
